# Comparative Phytochemical Constituents and Antioxidant Activity of Wild and Cultivated *Alepidea amatymbica* Eckl & Zeyh

**DOI:** 10.1155/2020/5808624

**Published:** 2020-04-13

**Authors:** Ramatsobane Maureen Mangoale, Anthony Jide Afolayan

**Affiliations:** Medicinal Plants and Economic Development (MPED) Research Centre, Department of Botany, University of Fort Hare, Alice 5700, South Africa

## Abstract

There is a need to scientifically validate the claim that wild species of medicinal plants are more potent than the cultivated plants. Thus, this study evaluated the phytochemical and antioxidant properties of wild and cultivated *Alepidea amatymbica*. Acetone, methanol, and water extracts of the rhizome of wild and cultivated *A. amatymbica* were evaluated for total phenol, flavonol, flavonoid, tannin, proanthocyanidin, saponin, and alkaloid contents using spectrophotometric methods. *In vitro* antioxidant activity was measured using 2,2-diphenyl-1-picrylhydrazyl (DPPH), 2,2′-azino-bis(3-ethylbenzothiazoline)-6-sulfonic acid (ABTS), ferric reducing antioxidant power (FRAP), nitric oxide (NO), and hydrogen peroxide assays. The wild extracts had higher phytochemical contents in most of the assays than cultivated extracts. Total phenol in the wild extracts ranged from 32.30 to 117.8 mg GAE/g with the acetone extracts having the highest content while the water extracts were the least. The range in the total phenol of the cultivated species was 66.46 to 98.44 mg GAE/g with the methanol extracts having the highest content while water extracts was the least. The flavonoid content ranged from 55.01 to 99.09 mg QE/g and from 48.65 to 67.32 mg QE/g for the wild and cultivated plants, respectively. The alkaloid contents ranged from 14.70 to 17.80% in the wild species while it ranged from 11.98 to 13.21% in the cultivated species. The wild species also showed higher antioxidant activities in most of the assays evaluated. This study has implications for both pharmacological and conservation purposes.

## 1. Introduction

The rhizomes of *Alepidea amatymbica* are widely used for the treatment of various diseases because of their effectiveness due to the presence of the bioactive compounds. Some of the phytochemicals isolated from dried rhizome of *A. amatymbica* include dehydro-16-kaurene-19-oic acid, *ent*-16-kauren-19-oic acid, wedelia seco-kaurenolide, 313-acetoxy, phenolic acid, and rosmarinic acid [1]. Phytochemical compounds such as phenolic acid and flavonoids have health benefits, and they are an important part of human diet [2]. These compounds have been reported in many studies as free radical scavengers against superoxide, lipid peroxyl, and hydroxyl radicals [3–5]. This, therefore, highlights many of their health-promoting functions as well as the prevention of diseases associated with oxidative damage of membranes, proteins, and deoxyribonucleic acid (DNA) [6]. The amount of polyphenolic constituents present in the plant affects its antioxidant activities [7]. Antioxidants are potential scavengers of free radicals in the body and protect the body against ill health and ravages of aging [8]. The use of natural antioxidants from plants has gained much attention as they are believed to be safer and have greater therapeutic activity than synthetic antioxidants [9].

According to the International Union for Conservation of Nature and Natural Resources (IUCN) red data list criteria of South Africa, *A. amatymbica* is considered vulnerable and under conservation in protected areas [10]. It is mostly harvested for medicinal purposes and is used as antibacterial, antifungal, anti-inflammatory, antihypertensive, anti-HIV, and diuretic agents [1]. It is overexploited, as it is commonly harvested for commercial purposes and sold in traditional markets [11]. The collection and trading of the plant products provide additional income for the rural poor people but, however, may result in overexploitation which could lead to its extinction [12].

Domestication of medicinal plants offers an excellent means of halting overexploitation [13]. As a conservation approach, *ex situ* cultivation is becoming a key element of modern conservation strategies [14]. Unfortunately, medicinal plant users perceive cultivated plants as less potent compared to wild ones [15], and lack of scientific evidence on the potency of cultivated medicinal plants remains a limiting factor. It is also reported that domestication of wild plants has to be done with extreme care because they may fail to synthesize the bioactive constituents following relocation [16]. Several studies have reported that the majority of pharmacologically important plant compounds are produced during defense mechanism when plants are subjected to stress and other secondary metabolites are produced due to the ability of plants to respond to chemical and physical stimuli [17, 18]. According to Bairu et al. [19], plants that are growing outside their natural environments will thus not produce desired bioactive compounds; hence, there is a need to evaluate this claim. Zheng et al. [20] also reported that the amount of phytochemicals in plants varies considerably from different ecological and climatic environments. No previous studies have been performed on the antioxidant and biological activities of *Alepidea amatymbica* grown in the wild and under cultivated conditions. Therefore, this study evaluated and compared phytochemical and antioxidant activity of the wild and cultivated *A. amatymbica*. The result of the study can provide useful information about the suitability of cultivated *A. amatymbica* rhizome as an alternative to wild species in terms of bioactive compounds.

## 2. Material and Methods

### 2.1. Collection and Preparation of Plant Material

The wild rhizome of *A. amatymbica* was obtained from its natural habitat in Emnyameni village (32°34′17.39^″^S; 27°6′55.16^″^E) in the Keiskammahoek area of the Eastern Cape Province, South Africa, in May 2014. The vegetation type is Amathole Montana grassland [21]. The cultivation of the plant was carried out in a greenhouse until flowering (May–December 2014) at Fort Cox College of Agriculture and Forestry. The rhizomes from both wild and cultivated *A. amatymbica* were collected at the flowering stage. Plants were authenticated at the Department of Botany in the University of Fort Hare, and voucher specimens Mau2015/04 and Mau2015/05 were deposited at the Giffen Herbarium of the university.

### 2.2. Reagents Used

The reagents used are as follows: methanol, acetone, aluminum chloride (AlCl_3_), quercetin dehydrate, tannic acid, Folin-Ciocalteu, sodium carbonate (Na_2_CO_3_), hydrochloric acid (HCL), diethyl ether, butanol, sodium chloride, vanillin, ammonium hydroxide (NH_4_(OH)_2_), azino-bis(3-ethylbenzthiazoline)-6-sulphonic acid (ABTS), 2,2-diphenyl-1-picrylhydrazyl (DPPH) radical scavenging activity, potassium persulfate, vitamin C, butylated hydroxytoluene (BHT), potassium ferricyanide (K_3_Fe(CN)_6_, trichloroacetic acid, ferrous chloride (FeCl)_3_, sodium nitroprusside, sulfonic acid, naphthylethylene diamine dichloride, acetic acid, ascorbic acid, butylated hydroxyl toluene, rutin, and hydrogen peroxide (H_2_O_2_). The chemicals used for this study were of standard grade purchased from Merck and Sigma-Aldrich (Gauteng Province, South Africa). Chemicals mentioned above were of analytical grade.

### 2.3. Preparation of the Plant Extracts

The rhizomes from wild and cultivated plants were rinsed with deionized water to remove soil particles and gently blotted with paper towel to remove water before chopping using a sterilized kitchen grater. Portions of grated wild and cultivated fresh *A. amatymbica* (200 g) were separately extracted in different solvents (acetone, water, and methanol) on an orbital shaker (Stuart Scientific Orbital SOI, Essex) for 24 hours. Each extract was filtered using a Buchner funnel and Whatman No. 1 filter paper. Water extract was frozen at 40°C and concentrated using a freeze dryer (VirTis BenchTop K, VirTis Co., Gardiner, NY) for 48 hours. Acetone and methanol extracts were concentrated to dryness under reduced pressure using rotary evaporator (Strike 202, Steroglass, Italy). Each extract was subsequently reconstituted in its solvent of extraction to give desired concentrations used for the various analyses.

### 2.4. Qualitative Phytochemical Screening

#### 2.4.1. Determination of Total Phenols

Total phenol content was determined using the Folin-Ciocalteu method described by Oyedemi et al. [22] using tannic acid as standard. Half a millilitre (0.5 mL) of the extracts (1 mg/mL) was mixed with 2.5 mL of 10% Folin-Ciocalteu solution and 2 mL of Na_2_CO_3_ (75% *w*/*v*). The mixture was vortexed for 30 seconds, and the resulting mixture was incubated at 40° C for 30 minutes for color development. Absorbance was measured at 765 nm using a UV spectrophotometer (AJI-CO_3_ UV-VIS). The total phenolic content of the sample was extrapolated from as mg/g of gallic acid standard (mg GAE/g) using the following derived equation based on the calibration curve of the standard: *Y* = 8.773*x* + 0.1956 (*R*^2^ = 0.9965), where *x* is the absorbance and *Y* is the gallic acid equivalent (mg GAE/g). mg GAE/g is derived from the equation *CV*/*m*, where “*C*” is the concentration as derived from the calibration curve equation in mg/mL, “*V*” is the volume of the extract used in the assay in mL, and “*m*” is the mass of the extract used in the assay in “g.”

#### 2.4.2. Determination of Total Flavonoids

Total flavonoids in *A. amatymbica* were quantified using the method of Arjamand et al. [23]. Half a millilitre (0.5 mL) of 2% AlCl_3_ in ethanol was added to 0.5 mL (1 mg/mL) of the sample solution and incubated for 1 hour at room temperature, after which the absorbance was measured at 420 nm. The extracted sample was evaluated at a final concentration of 0.1 mg/mL, and the flavonoid content was calculated as quercetin (mg/g) equivalents using the following equation based on the calibration curve: *Y* = 0.025*x* + 0.1335 (*R*^2^ = 0.9812), and expressed as mg of quercetin equivalent (QE/g).

#### 2.4.3. Determination of Total Flavonols

Total flavonol content was estimated using the method of Mbabie et al. [24]. Two millilitres (2 mL) of plant extracts (1 mg/mL) was mixed with 2 mL of AlCl_3_ prepared in ethanol and 3 mL of 50 g/L sodium acetate solution. The absorption at 440 nm was measured after 2.5-hour incubation at 20°C. The total flavonol content was calculated as quercetin (mg/g) using the derived equation based on the calibration curve *Y* = 0.0255*x* (*R*^2^ = 0.9812), where *Y* is the quercetin equivalent (mg QE/g) and *x* is the absorbance. The formula used was *CV*/*m*, as described above for total phenols.

#### 2.4.4. Determination of Total Proanthocyanidin

Total proanthocyanidin was estimated as described by Kibiti and Afolayan [3]. Half a millilitre of 0.1 mg/mL of extract solution was mixed with 3 mL of 4% vanillin-methanol solution and 1.5 mL hydrochloric acid (HCL) and vortexed. The resulting mixture was allowed to stand for 15 minutes at room temperature followed by the measurement of the absorbance at 500 nm. The experiment was done in triplicate. Proanthocyanidin content was evaluated using the calibration curve equation: *Y* = 0.9038*x* + 0.0449 (*R*^2^ = 0.9951), and was expressed as mg catechin equivalent (CE)/g using the formula from the equation *CV*/*m*, where “*C*” is the concentration as derived from the calibration curve equation in mg/mL, “*V*” is the volume of the extract used in the assay in mL, and “*m*” is the mass of the extract used in the assay in “g”.

#### 2.4.5. Determination of Total Tannin Content

Tannin content was estimated according to Sowunmi and Afolayan [4] with slight modifications. 0.20 g of the sample was added to 20 mL of 50% methanol. This mixture was shaken thoroughly and placed in a water bath at 80°C for 1 hour to ensure uniform mixing. The extract was filtered into a 100 mL volumetric flask, followed by the addition of 20 mL distilled water, 2.5 mL Folin-Ciocalteu reagent, and 10 mL of 17% aqueous Na_2_CO_3_. The mixture was made up to 100 mL with distilled water and allowed to stand for 20 minutes. The development of a bluish green color was observed at the end of the reaction mixture of the different concentration ranges from 0 to 10 ppm. The absorbance of the tannic acid standard solutions as well as the sample was measured at 760 nm. Results were expressed as mg/g of tannic acid equivalent using the calibration curve: *Y* = 0.0593*x* + 0.0485 (*R*^2^ = 0.9826), where *x* is the absorbance and *Y* is the tannic acid equivalent (mg/g).

#### 2.4.6. Determination of the Saponin Content

Saponin content was determined as described by Otunola and Afolayan [25]. Five grams of plant materials was dispersed in 50 mL of 20% *v*/*v* ethanol prepared in distilled water. The mixture was heated in a hot water bath at 55°C for 4 hours with continuous stirring. The mixture was filtered, and the residue was reextracted with another 50 mL of 20% aqueous ethanol. The volume was reduced to 20 mL in a hot water bath at 100° C. The concentrate was transferred to a separating funnel, and 10 mL diethyl ether was added. The concentrated solution obtained was shaken vigorously in a separating funnel, and ether layer was discarded, while the aqueous layer was retained. The process was repeated twice for purification purposes, and 20 mL of butanol was added to the aqueous layer. The n-butanol extracts were washed twice with 10 mL of 5% (*w*/*v*) aqueous sodium chloride, and the mixture was heated to evaporation on a hot water bath, then oven dried at 40°C to constant weight. The percentage saponin content of the sample was calculated using the formula

(1) $$\begin{array}{c} \%\text{Saponin content}=\frac{\text{weight of residue}}{\text{weight of original sample}}\times 100. \end{array}$$

#### 2.4.7. Determination of Alkaloids

Alkaloid contents were quantitatively determined according to Olajuyigbe and Afolayan [26]. Five grams of fresh rhizome samples was soaked in 200 mL of 10% acetic acid in ethanol. The mixture was covered and allowed to stand for 4 hours. The filtrate was concentrated in a water bath to a quarter of its original volume. Concentrated NH_4_(OH)_2_ was added dropwise to the filtrate until precipitation was completed, and the whole solution was allowed to settle. The collected precipitates were washed with dilute ammonium hydroxide then filtered again. The residue collected was dried and weighed. The alkaloid content was determined using the formula

(2) $$\begin{array}{c} \%\text{alkaloid}=\frac{\text{weight of precipitate}}{\text{weight of original sample}}\times 100. \end{array}$$

### 2.5. Determination of In Vitro Antioxidant Activity

The antioxidant activities of water, acetone, and methanol of cultivated and wild *A. amatymbica* were determined using 2,2′-azino-bis(3-ethylbenzthiazoline-6-sulphonic acid (ABTS), 2,2-diphenyl-1-picrylhydrazyl (DPPH) radical scavenging activity, ferric reducing power, nitric oxide, and hydrogen peroxide inhibitory assays.

#### 2.5.1. 2,2′-Azino-bis(3-ethylbenzthiazoline)-6-sulphonic Acid (ABTS) Radical Scavenging Assay

The method of Wintola and Afolayan [27] was adopted for the determination of ABTS activity of the plant extract. The ABTS radical was generated by allowing a mixture of 7 mM ABTS and 2.4 mM potassium persulfate in the same ratio to react in the dark for overnight at room temperature. The solution was then diluted by mixing 1 mL ABTS in 60 mL of methanol to obtain the absorbance of 0.706 ± 0.001 units at 734 nm using a spectrophotometer. ABTS solution was freshly prepared for the assay. One millilitre (1 mL) of different concentrations (0.025, 0.05, 0.1, 0.2, and 0.5 mg/mL) of the extracts and standard drugs was allowed to react with 1 mL of ABTS radical in the dark for 7 minutes. The absorbance was taken using a spectrophotometer at 734 nm. The percentage inhibition of ABTS by the plant extract was compared with that of vitamin C and BHT as standards. The percentage inhibition of ABTS was calculated as

(3) $$\begin{array}{c} \%\text{ABTS scavenging activity}=\left[\left(\text{Abs }\left(\text{control}\right)-\text{Abs }\left(\text{sample}\right)\right)/\left(\text{Abs control}\right)\right]\times 100. \end{array}$$

where Abs (control) is the absorbance of ABTS+radical+methanol and Abs (sample) is the absorbance of ABTS+radical+sample extract or standard.

#### 2.5.2. 2,2-Diphenyl-1-picrylhydrazyl (DPPH) Radical Scavenging Activity

The scavenging activity of the plant extract against DPPH was determined according to the method described by Otang et al. [5]. A solution of 0.135 mM DPPH in methanol was prepared, and 1 mL of this solution was mixed with different concentrations (0.025, 0.05, 0.1, 0.2, and 0.5 mg/mL) of various extracts of *A. amatymbica* and standard antioxidants (vitamin C and BHT). The reaction mixture was vortexed thoroughly and left in the dark at room temperature for 30 minutes and absorbance measured at 517 nm. The ability of the plant extracts to scavenge DPPH was calculated as

(4) $$\begin{array}{c} \%\text{DPPH inhibition }\left[\left(\text{Abs }\left(\text{control}\right)–\text{Abs }\left(\text{sample}\right)\right)/\left(\text{Abs control}\right)\right]\times 100, \end{array}$$

where Abs (control) is the absorbance of DPPH radical+methanol and Abs (sample) is the absorbance of DPPH radical+sample extracts/standard.

#### 2.5.3. Determination of Ferric Reducing Antioxidant Power (FRAP)

The reducing power of *A. amatymbica* extract was evaluated according to the method described by Idris et al. [28] with a slight modification. The mixture containing 2.5 mL of 0.2 M phosphate buffer (pH 6.6) and 2.5 mL of potassium ferricyanide (K_3_Fe(CN)_6_) (1%) was added to 1 mL of each extract at different concentrations (0.025, 0.05, 0.1, 0.2, and 0.5 mg/mL). The mixture was incubated at 50°C for 20 minutes. After incubation, 2.5 mL of trichloroacetic acid (10%) was added to terminate the reaction. The mixture was centrifuged at 3000 rpm for 10 minutes. The supernatant (2.5 mL) was collected, and it was mixed with distilled water (2.5 mL) and 0.5 mL FeCl_3_ (0.1%). The absorbance of the reaction mixture was then measured at 700 nm against the blank sample (without extract). Ascorbic acid and butylated hydroxyl toluene solutions were used as positive controls. Increase in absorbance of the reaction mixture indicated increased reducing power.

#### 2.5.4. Nitric Oxide (NO) Radical Scavenging Activity Assay

Nitric oxide scavenging activity was determined using the method described by Ebrahimzadeh et al. [29]. Two millilitres (2 mL) of 10 mM sodium nitroprusside dissolved in 0.5 mL phosphate-buffered saline (pH 7.4) was mixed with 0.5 mL of plant extract or vitamin C or rutin at various concentrations (0.025, 0.05, 0.1, 0.2, and 0.5 mg/mL). The resulting mixture was incubated at 25°C for 2.5 hours. After incubation, 0.5 mL of the reaction mixture was taken and mixed with 0.5 mL of Griess reagent (containing 1.0 mL of 0.33% sulfonic acid in 20% glacial acetic acid) and was incubated for 10 minutes at room temperature with 1 mL of naphthylethylene diamine dichloride (0.1% *w*/*v*). The mixture was incubated at room temperature for 30 minutes, and the absorbance measured at 540 nm. The inhibition of nitric oxide radical was calculated as

(5) $$\begin{array}{c} \left[\left(\text{Abs }\left(\text{control}\right)-\text{Abs }\left(\text{sample}\right)\right)/\left(\text{Abs }\left(\text{control}\right)\right)\right]\times 100. \end{array}$$

where Abs (control) is the absorbance of NO radical+methanol and Abs (sample) is the absorbance of NO radical+sample extract or standard.

#### 2.5.5. Hydrogen Peroxide Scavenging Activity Assay

The ability of the plant extract to scavenge hydrogen peroxide was determined by the method of Kibiti and Afolayan [3]. Two millilitres (2 mL) of plant extracts prepared at various concentrations (0.025, 0.05, 0.1, 0.2, and 0.5 mg/mL) was mixed with 6 mL of 4 mM H_2_O_2_ solution in phosphate buffer (0.1 M pH 7.4). The mixture was incubated for 10 minutes at room temperature. The absorbance of the solution was measured at 230 nm against the blank solution containing the plant extracts without H_2_O_2_. The result obtained was compared with standard ascorbic acid. Percentage inhibition of H_2_O_2_ = [(Abs (control) − Abs (sample))/(Abs (control))] × 100, where Abs (control) is the absorbance of the control and Abs (sample) is the absorbance of the extracts/standard.

### 2.6. Data Analysis

All data were expressed as mean ± standard deviation (SD) of three replications. One-way analysis of variance (ANOVA) was used to compare the differences between the cultivated and wild *A. amatymbica* samples. Where applicable, data were subjected to statistical analysis using SAS (Statistical Analysis System) package. Mean separation was done using Fischer's LSD where the data showed significance (*p* < 0.05). The concentrations required to attain 50% radical scavenging activity (IC_50_) was determined from regression lines with 95% confidence level.

## 3. Results

### 3.1. Quantification of Phytochemicals

Phytochemical constituents of wild and cultivated *A. amatymbica* revealed the presence of tannins, saponins, alkaloids, proathocyanidins, flavonoids, flavonols, and total phenolics (Table 1). The phytochemicals obtained from the plant extracts varied greatly among the three solvents, in both wild and cultivated plants. Wild and cultivated plants showed a significant difference (*p* < 0.05) in tannin content for both acetone and methanol extracts but not in water extracts. The methanol and acetone extracts of wild and cultivated plants showed a significant difference (*p* < 0.05) in the alkaloid contents except in water extracts. Saponin contents in both wild and cultivated extracts of *A. amatymbica* showed a significant difference (*p* < 0.05) in all solvents. Methanol and water extracts of wild plants showed a significantly higher (*p* < 0.05) saponin content of (34.47 and 12.80 mg/g, respectively) except in acetone extracts. The total phenolic content of wild and cultivated plants showed a significance difference (*p* < 0.05) in acetone and water extracts only. Methanol and water extracts of cultivated plants had higher total phenolic content of 98.44 and 66.46 mg/g, respectively. Wild *A. amatymbica* extracts exhibited significantly higher (*p* < 0.05) total flavonols, flavonoids, and proanthocyanidin contents in all solvents compared to cultivated plants.

### 3.2. Ferric Antioxidant Reducing Power (FRAP)

**Figure 1:**
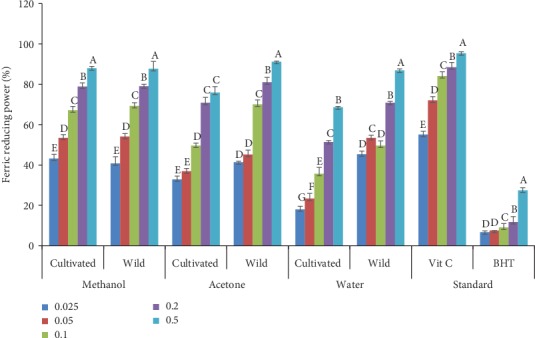
Reducing power of acetone, methanol, and water extracts of wild and cultivated *A. amatymbica*. Bar graphs with different letters within the same solvents are significantly different (*p* < 0.05); wild = wild *A. amatymbica*; cultivated = cultivated *A. amatymbica*.

The reducing power of the wild and cultivated *A. amatymbica* from the three solvent extracts and standard drugs is shown in Figure 1. The reducing power of the solvent extracts on ferric to ferrous gradually increased with increase in concentration. The reducing capacity of methanol extracts of wild and cultivated plants within a concentration showed no significant difference (*p* > 0.05) in all concentrations tested. Acetone extracts of the wild plants had a significantly higher mean (*p* < 0.05) reducing power than acetone extracts of the cultivated plants. Significantly higher (*p* < 0.05) reducing powers were also observed in water extracts of the wild plants at all concentrations assayed.

### 3.3. Hydrogen Peroxide Inhibition

**Figure 2:**
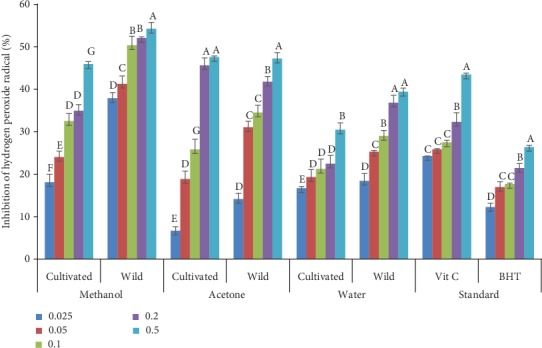
Hydrogen peroxide radical scavenging activities of acetone, methanol, and water extracts of wild and cultivated extracts of *A. amatymbica* rhizome. Bars with different superscript letters within the same solvent are significantly different (*p* < 0.05); wild = wild *A. amatymbica*; cultivated = cultivated *A. amatymbica*.

The hydrogen peroxide radical inhibition activities of wild and cultivated *A. amatymbica* are presented in Figure 2. There was a concentration-dependent increase in inhibition in the extracts assayed. The methanol extracts of both wild and cultivated plants showed a significant difference (*p* < 0.05) at all concentrations. The methanol extract of the wild plants showed significantly higher mean (*p* < 0.05) radical inhibition at all concentrations. There was also a significant difference (*p* < 0.05) in radical inhibition by the acetone extracts of the cultivated and wild plant at all concentrations except at 0.5 mg/mL. Acetone extracts of the wild plants had significantly higher (*p* < 0.05) scavenging activity of 14.15, 31.04, and 34.51% at 0.025, 0.05, and 0.1 mg/mL, respectively, except at 0.2 mg/mL. The water extracts of the wild plants had significantly higher radical inhibition compared to water extracts of the cultivated plants.

### 3.4. ABTS Radical Scavenging Activity

**Figure 3:**
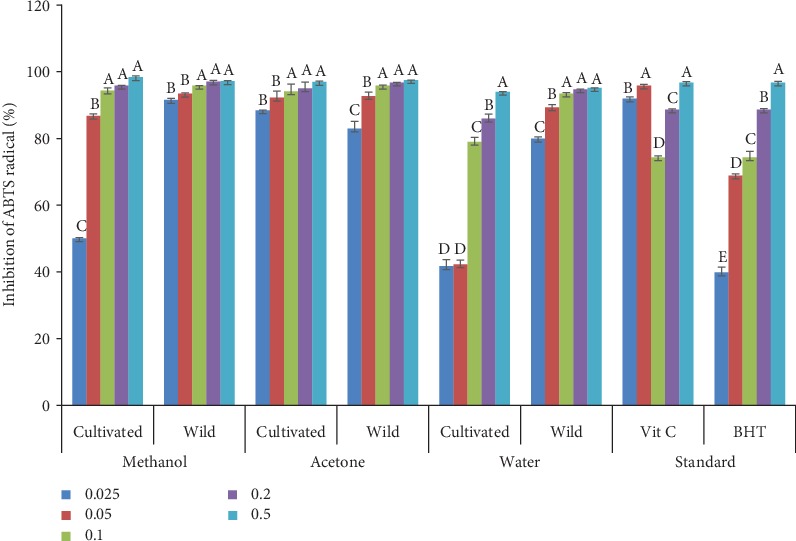
ABTS radical scavenging activity of acetone, methanol, and water extracts from wild and cultivated extracts of *A. amatymbica* rhizome obtained from different solvents. Bar graphs with different superscript letters within the same solvent are significantly different (*p* < 0.05); wild = wild *A. amatymbica*; cultivated = cultivated *A. amatymbica*.

The results of ABTS radical scavenging activity of wild and cultivated *A. amatymbica* at various concentrations are shown in Figure 3. There was a concentration-dependent response in this assay. All the solvent extracts showed great ABTS radical scavenging activity at very low concentrations. There was no significant difference (*p* > 0.05) in radical scavenging activity in the methanol extracts of the wild and cultivated plants except at a concentration of 0.025 mg/mL. Methanol extracts of the wild plants had a significantly higher (*p* < 0.05) radical scavenging activity of 91.55% at 0.025 mg/mL. The acetone extracts of both wild and cultivated plants showed no significant difference (*p* > 0.05) at all concentrations except at 0.025 mg/mL. Acetone extracts of the cultivated plants showed significantly higher ABTS inhibition of 88.45%. A significant difference (*p* < 0.05) was observed in the water extracts of wild and cultivated plants at all concentrations but not at 0.5 mg/mL. Water extracts of the wild plants exhibited higher radical scavenging activity of 79.96, 89.37, 93.51, and 94.74% at 0.025, 0.05, 0.1, and 0.2 mg/mL concentrations, respectively, compared to those of the cultivated plants.

### 3.5. DPPH Radical Scavenging Activity

**Figure 4:**
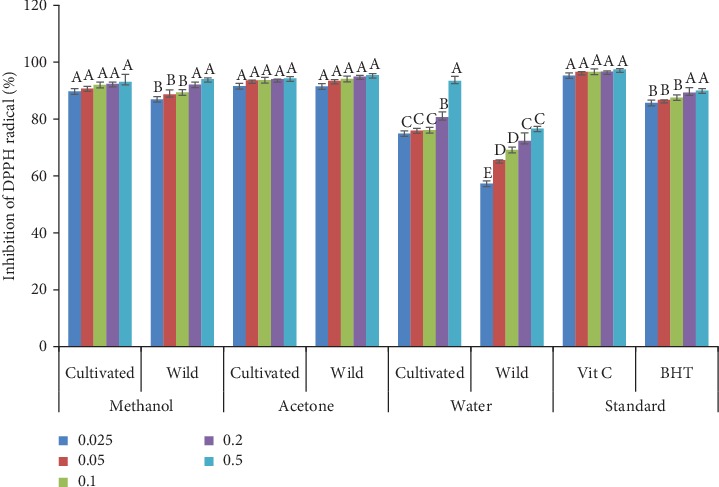
DPPH radical scavenging activity of acetone, methanol, and water extracts of wild and cultivated *A. amatymbica*. Bars with different letters within the same solvents are significantly different (*p* < 0.05).

The DPPH radical scavenging activity of the different solvent extracts of wild and cultivated *A. amatymbica* is compared to that of known antioxidants (vitamin C and BHT), and their respective concentrations and are presented in Figure 4. The DPPH radical scavenging potential in all solvent extracts and BHT significantly increased with increasing concentrations in both wild and cultivated plants. The methanol extracts of wild and cultivated plants showed a significant difference (*p* < 0.05) at all concentrations except at 0.2 mg/mL and 0.5 mg/mL. The methanol extract of the cultivated plants exhibited a higher radical scavenging activity of 89.4, 90.77 and 92.05% at 0.025, 0.05 and 0.1 mg/mL concentrations, respectively. No significant difference (*p* > 0.05) was observed on the DPPH radical scavenging activity of acetone extracts of the wild and cultivated plants. The water extracts of wild and cultivated plants showed a significance difference (*p* < 0.05) at all concentrations. Water extracts of cultivated plants showed a significantly higher (*p* < 0.05) radical scavenging activity.

### 3.6. Nitric Oxide (NO) Inhibition

**Figure 5:**
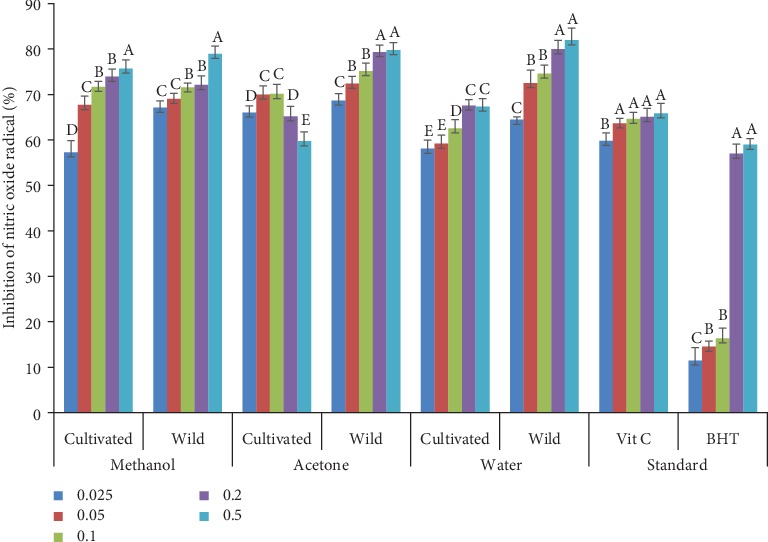
Nitric oxide radical scavenging activity of the acetone, methanol, and water extracts from wild and cultivated *A. amatymbica* rhizomes. Bar graphs with different letters within the same solvents are significantly different (*p* < 0.05). Wild = wild *A. amatymbica*; cultivated = cultivated *A. amatymbica*.

The inhibitory effect of various extracts of wild and cultivated *A. amatymbica* comparable to the standards (BHT and vitamin C) is presented in Figure 5. Extracts from three solvents demonstrated a strong scavenging activity against nitric oxide radical compared to standards. There was no significant difference (*p* > 0.05) in the radical scavenging activity of the methanol extracts of wild and cultivated plants at all concentrations excepts at 0.025 mg/mL. Methanol extracts of the wild plants had a significantly higher (*p* < 0.05) scavenging activity of 67.7% compared to methanol extracts of the cultivated plants. The acetone extracts of wild and cultivated plants showed a significant difference (*p* < 0.05) in radical scavenging activity at all concentrations assayed. Acetone extracts of the wild plants had a significantly higher mean inhibition at all concentrations. Higher significant (*p* < 0.05) inhibition was also observed in the water extracts of wild plants compared to cultivated plants at all concentrations.

### 3.7. Effects of Wild and Cultivated *A. amatymbica* Extracts and Standards (BHT and Vitamin C) at Their Respective Concentrations That Scavenged 50% (IC_50_) of the Radicals

The inhibition of ferric reducing power, hydrogen peroxide, ABTS, and DPPH by all extracts and standards at their respective concentrations that scavenged 50% (IC_50_) of their radicals is shown in Table 2. The ferric reducing power scavenging activity of the extracts and the standard drugs based on the IC_50_ was in the decreasing order: vitamin C (standard) > wild (water) > wild (methanol) > cultivated (methanol) > wild (acetone) > cultivated (acetone) > cultivated (water) > BHT (standard). The wild plants showed a better reducing IC_50_ than the cultivated plants. The hydrogen peroxide scavenging activity as recorded from the IC_50_ values is in the following order: wild (methanol) > wild (acetone) > cultivated (acetone) > vitamin C (standard) > wild (water) > cultivated (methanol) > cultivated (water) > BHT (standard). However, water extracts of the cultivated plants and BHT showed no activity against hydrogen peroxide.

The IC_50_ values showed ABTS radical scavenging potential of all the extracts, and the standards were in the following order: vitamin C (standard) > wild (methanol) > cultivated (acetone) > wild (acetone) > wild (water) > cultivated (methanol) > cultivated (water) > BHT (standard). Methanol extracts of the wild plants, acetone extracts of cultivated plants, and vitamin C showed the higher potential of ABTS as evaluated by the IC_50_ which was greater than the least concentration evaluated. Methanol extracts and acetone extracts of the wild plants and acetone extracts of the cultivated plants showed higher potential of DPPH with IC_50_ which was greater than the least concentration evaluated. The DPPH radical scavenging potential followed this trend: BHT > wild (methanol) > cultivated (acetone) > wild (acetone) > cultivated (water), wild (water) > vitamin C. The acetone extracts of the cultivated plants showed no activity on the scavenging of the nitric oxide. The nitric oxide radical scavenging potential of all the extracts and the standards based on the IC_50_ was in the decreasing order: BHT, wild (methanol) > wild (water) >cultivated (water) >wild (acetone) > cultivated (methanol) > vitamin C > BHT > cultivated (acetone).

## 4. Discussion

Phytochemical contents varied greatly among the three solvents in both wild and cultivated plants. This is an indication that the solvents have different extracting abilities for phytochemicals. It was observed that the phytochemical constituents in the wild plants were higher than those in the cultivated plants. The harsh seasonal variations may explain the differences in the concentration of bioactive compounds between the wild and cultivated *A. amatymbica* [30, 31]. The differences may be due to agronomic practices which may influence the environmental conditions, nutrients in the soil, and other parameters that may alter the growth rate and metabolic activity of the plant [32]. Arjamand et al. [23], in their studies on wild and cultivated *Satureja bachtiarica*, reported agromorphological divergence which may have contributed to great variability in the value of the essential oil phytochemical constituents of the plant.

In this research, quantitative variability in the flavonoid, tannin, flavonol, and proanthocyanidin contents of the wild and cultivated *A. amatymbica* may be related to differences in geographical locations, climatic conditions, soil type, and natural or anthropogenic disturbances. It was previously reported that the wild state of the plant is usually in a shady and moist habitat but the cultivated plant may be inconsistently shaded and other environmental conditions may not be adequately simulated unlike the ones in the wild; hence there may be phytochemical variations [31] as shown in the results of this research. According to Ashashri et al. [33] and Tajalli et al. [34], the exposure of plants to harsh environmental conditions including insects and infections is known to enhance the productions of flavonols and flavonoids. These phytochemicals are for self-protection. Hence, significantly higher concentrations of flavonoids and flavonols were observed in wild *A. amatymbica*. Lower concentrations of flavonols, flavonoids, tannins, and flavonoids in cultivated plants might be due to the plants being unable to produce desired bioactive compounds under controlled conditions. Similar results were also reported by Sefidkon et al. [35], who observed that cultivated plants produced lower levels of secondary metabolites due to monoculture cultivation conditions. Several studies have reported that majority of pharmacologically important compounds of plant origin are products of defense to protect plants against stress, insects, herbivores, and diseases [16–18]. The presence of flavonoids in both wild and cultivated plants may show the ability of this plant to scavenge free radicals [36]. The higher content of proanthocyanidin observed in this study may validate the claim of medicinal plant users about antihypertensive properties of *A. amatymbica* which was also reported by Wintola and Afolayan [1].

The nonsignificant difference in alkaloid and tannin contents observed in the water extracts of the wild and cultivated plants might be due to the polarity of solvent used; hence, the significant difference observed in acetone and methanol extracts of wild and cultivated plants may depend on the ability of the solvents to extract some key constituents. (Umpathy et al. [37]). The variation in phytochemical as shown in Table 2, thus, confirmed that variation between wild and cultivated plants may be due to the influence of agronomic parameters such as shading or sunlight exposure and water supply which had impact on chemical composition. The findings of this study were in line with Ahmed et al. [38] in which bioactive compounds from herbal medicine may be affected by physiological response (circadian rhythm and phenology) of plants and agronomic conditions. It has also been reported that growing conditions, such as warm weather, increase the synthesis of secondary metabolites but wet weather conditions can inhibit alkaloid production in many species [19]. Alkaloids have been used medically as analgesic and antiseptic [39]. The presence of alkaloid in both wild and cultivated *A. amatymbica* shows that the plant could be a potential source of antispasmodic, antimalarial, antiseptic, and antibacterial agents [40]. This could explain the use of *A. amatymbica* by traditional healers for the treatment of the above ailments. The presence of tannins in wild and cultivated *A. amatymbica* makes the plant a good source of tannin and could be useful for the prevention of cancer as well as treatment of inflamed ulcerated tissue. It can also act as a natural antibiotic by preventing lipid peroxidation [41, 42].

The significantly higher phenolic content in the methanol and water extracts of cultivated *A. amatymbica* might indicate a certain stress parameter that may not be justified in this research. However, it may have been attributed to growing *A. amatymbica* outside its habitat. Similar results were also obtained by Soriano-Melgar et al. [43], who demonstrated that domestication process and cultivation increased some phytochemicals and antioxidant activities of *Turnera diffusa*. Willd. ex Schult. It has been reported that phenolics inactivate iron ions by chelating and additionally suppressing the superoxide-driven Fenton reaction, which is believed to be the most important source of reactive oxygen ions [44]. The results from this study suggest that high phenolic content in cultivated *A. amatymbica* could have pharmacological effects which may also be attributed to agromorphological divergence. These results also suggest that relocating these plants from the wild to nursery did not result in the loss of phenols. Therefore, results further confirm that the similarity in some of phytochemicals between the wild and cultivated plants may be helpful for the domestication of *A. amatymbica* by medicinal plant users.

The nonsignificant difference observed in ferric reducing power of the methanol extracts of wild and cultivated *A. amatymbica* might be to the high amount of phenolic content in the plants. Significantly higher ferric reducing power observed in acetone and water extracts of wild *A. amatymbica* at all concentrations may be due to the higher content of phytochemical in the wild plants. Several authors have reported that antioxidant activity expression is a consequence of the synergism between different phytochemical compounds and can be attributed to constituents [27, 45, 46]. The significant influence of environmental conditions on the phytochemicals and consequently to ferric reducing power of wild and cultivated *A. amatymbica* may be indicating the important role played by the environment, ultraviolet (UV) radiation, temperature, water stress, and mineral nutrient availability [47]. Amounts of phytochemical constituents found in both wild and cultivated *A. amatymbica* correlated with antioxidant activity which confirms that they are contributing factors towards its antioxidant properties [48].

Hydrogen peroxide is an important reactive oxygen species (ROS) which has the ability to penetrate biological molecules and convert toxic to hydrophilic and breakdown to hydroxyl radical in the cell. However, the hydroxyl radical generated in the cell has been reported to induce breakage in DNA strand and chemical changes in the deoxyribose and nitrogenous bases; hence, hydroxyl radicals generated are degraded by the liver [49]. This research revealed that wild *A. amatymbica* exhibited a high percentage of hydrogen peroxide radical scavenging activity and this may be attributed to the higher flavonol, flavonoid, and proanthocyanidin contents in the wild plant compared to cultivated ones. This result is in agreement with the findings of Motlhanka [46], who reported that the cultivation has reduced antioxidant activity. It has been reported that drought and salt stress in a natural environment cause production of reactive oxygen species (ROS) within the plant resulting in an increased level of secondary metabolites, including phytochemicals. Hence, wild plants exhibited higher hydrogen peroxide radical inhibition [50]. The reduction in the antioxidant efficacy of the cultivated *A. amatymbica* could be a reason to depend more on the wild species for therapy.

Significance difference in ABTS^+^ inhibition observed in acetone and methanol extracts of wild *A. amatymbica* and methanol extracts of cultivated *A. amatymbica* at 0.025 mg/mL may be due to the lesser phytoconstituent content in the cultivated plants. This study reflected the capacity of wild and cultivated *A. amatymbica* to inhibit or quench free radicals. The ABTS radical scavenging potential exhibited by wild plant extracts could be attributed to polyphenolic compounds as earlier shown in the results. This agrees with several reports of plants which show antioxidant activity with ABTS [22, 25, 51]. The ability of the extracts to scavenge free radicals supports the folkloric use of the plant as a useful therapeutic agent for treating radical-related pathological damages. Also, it confirms the fact that cultivation of *A. amatymbica* did not affect the ability of the plant to inhibit free radicals.

The scavenging ability of the stable DPPH radical is widely used in evaluating the free radical scavenging ability of various compounds [25]. It was observed that DPPH radical scavenging potential in all solvent extracts and BHT significantly increased with increasing concentrations in both wild and cultivated plants. The research revealed that water extracts of the cultivated *A. amatymbica* exhibited significantly higher DPPH radical inhibition which may be due to the significantly higher phenolic compounds present in the water extracts of the cultivated plants. Similar results were reported by Masondo et al. [52] about the potency of cultivated *Bowiea volubilis* in traditional medicine. The similarity in DPPH radical scavenging activity of acetone extracts of wild and cultivated plants in this study could be attributed to the presence of other bioactive agents capable of donating electrons. This result indicates the ethnopharmacological potential of cultivated *A. amatymbica* in traditional medicine.

Nitric oxide is also an important cellular signaling molecule needed in low amounts to protect some vital organs in the human body, especially the liver and heart, from ischemic damage and hypertension. However, the overproduction of this molecule may cause inflammation and pathogenesis of various human diseases such as cardiovascular diseases which may be detrimental to human health. Higher nitric oxide observed in wild *A. amatymbica* might be due to the fact that the therapeutic properties of herbal medicines are biosynthesized by plants under particular conditions of stress and competition in their natural environments [53]. Hence, extracts of the cultivated plants had a lower nitric oxide inhibition activity. It was also observed that extracts of wild and cultivated plants showed good nitric oxide scavenging activity as compared to those of the standards. The nitric oxide inhibiting ability of the plant extracts could partially justify the use of wild plants in the treatment of oxidative-induced ailments due to its higher scavenging activity.

## 5. Conclusion

There were significant differences among compositions and antioxidant activities of studied extracts. The wild plants exhibited superior levels of phytochemical and antioxidant activities than cultivated *A. amatymbica*. Even though cultivated plants of *A. amatymbica* showed lower levels of proanthocyanidin, total flavonols, total flavonoids, alkaloids, and tannin, this however showed that the potential of the plant has been used in an antioxidant agent as the IC_50_ showed activity at the lowest concentrations tested. Therefore, this IC_50_ gives an indication that cultivated *A. amatymbica* can be effectively used in traditional medicine rather than relying on the rhizome from the wild populations alone. Based on the presented results, the rareness of natural antioxidant medicinal plants due to the cost and unavailability of the wild plants could be overcome by using cultivated species. Domestication of these wild plants coupled with extensive qualitative profiling to validate consistency in phytochemical composition can reduce the pressure on the wild populations.

## Figures and Tables

**Table 1:** Phytochemical constituents of acetone, water, and methanol extracts from wild and cultivated *Alepidea amatymbica* rhizomes.

Phytochemical constituents	Extracts of wild *A. amatymbica*			Extracts of cultivated *A. amatymbica*		
	Acetone	Methanol	Water	Acetone	Methanol	Water
Tannin (mg/g)	59.70^a^ ± 0.417	62.40^a^ ± 0.721	11.67^c^ ± 0.304	39.70^b^ ± 0.143	41.30^b^ ± 0.21	9.80^c^ ± 0.18
Alkaloids (%)	16.12^a^ ± 0.03	17.80^a^ ± 0.015	14.70^b^ ± 0.04	12.39^c^ ± 0.02	11.98^c^ ± 0.14	13.21^b^ ± 0.06
Saponin (%)	10.97^c^ ± 0.102	34.47^a^ ± 0.87	12.80^b^ ± 0.66	12.0^b^ ± 0.35	12.20^b^ ± 0.30	10.81^c^ ± 0.82
Total phenolics (mg GAE/g)	117.8^a^ ± 8.45	97.81^b^ ± 7.71	32.30^d^ ± 3.43	67.01^c^ ± 3.27	98.44^b^ ± 6.08	66.46^c^ ± 5.29
Total flavonols (mg QE/mg)	24.13^c^ ± 7.1	68.67^a^ ± 8.13	18.32^d^ ± 0.30	21.91^d^ ± 2.56	58.97^b^ ± 5.91	10.61^e^ ± 1.14
Total flavonoids (mg QE/mg)	97.33^a^ ± 7.20	99.09^a^ ± 7.20	55.01^c^ ± 6.51	59.66^c^ ± 0.17	67.32^b^ ± 4.1	48.65^d^ ± 7.74
Total proanthocyanidin (mg CE/g)	262.7^b^ ± 4.52	325.7^a^ ± 8.70	244.4^c^ ± 6.28	144.7^d^ ± 4.51	243.4^c^ ± 5.30	153.4^d^ ± 1.37

Data are expressed as means of triplicate (*n* = 3, mean ± standard deviation); means with different letters or superscripts are significantly different (a>b>c). Means with different superscripts or letters are significantly different (*p* < 0.05) in the different plants (wild and cultivated) but similar extracts or solvents. GAE: gallic acid equivalent; QE: quercetin equivalent; CE: catechin equivalent.

**Table 2:** IC _50_ values of acetone, methanol, and water fractions of wild and cultivated of *A. amatymbica* rhizomes and standards.

Activity samples	Wild *A. amatymbica*										Cultivated *A. amatymbica*									
	A		B		C		D		E		A		B		C		D		E	
	IC_50_^a^	*R* ^2^ ^b^	IC_50_^a^	*R* ^2^ ^b^	IC_50_^a^	*R* ^2^ ^b^	IC_50_^a^	*R* ^2^ ^b^	IC_50_^a^	*R* ^2^ ^b^	IC_50_^a^	*R* ^2^ ^b^	IC_50_^a^	*R* ^2^ ^b^	IC_50_^a^	*R* ^2^ ^b^	IC_50_^a^	*R* ^2^ ^b^	IC_50_^a^	*R* ^2^ ^b^
Acetone extract	0.0795	0.9972	0.5594	0.9694	0.00062	0.9191	^∗∗∗∗∗^	0.9422	0.00060	0.994	0.10483	0.998	0.59143	0.9947	^∗∗∗∗∗^	0.9631	^∗∗∗∗∗^	0.963	NA	NA
Water extract	0.03767	0.9928	2.266992	0.9887	0.00106	0.952	0.00531	0.9489	0.00196	0.956	0.19487	0.9983	NA	0.9981	0.03123	0.952	0.0009	0.096	0.0023	0.983
Methanol extract	0.03795	0.9722	0.099	0.9348	^∗∗∗∗∗^	0.9876	^∗∗∗∗∗^	0.9987	0.00041	0.99	0.03826	0.9972	0.738276	0.995	0.0249	0.9876	^∗∗∗∗∗^	0.988	0.0072	0.89
Vitamin C	0.01258	0.9547	0.658756	0.9971	^∗∗∗∗∗^	^∗∗∗∗∗^	0.6534	0.825	0.0156	0.87	0.01258	0.9547	0.658756	0.9971	^∗∗∗∗∗^	^∗∗∗∗∗^	0.6534	0.825	0.0156	0.87
BHT	2.7057	0.9972	NA	0.9918	0.03768	0.9981	^∗∗∗∗∗^	0.975	1.5255	0.964	2.7057	0.9972	NA	0.9918	0.03768	0.9981	^∗∗∗∗∗^	0.975	1.5255	0.964

A: ferric reducing power; B: hydrogen peroxide scavenging activity; C: ABTS scavenging activity; D: DPPH scavenging activity; E: nitric oxide scavenging activity. ^a^IC_50_ is defined as the concentration (mg/mL) sufficient to obtain 50% of maximum scavenging capacity. ^b^Coefficient of determination. Values obtained from regression lines with 95% confidence level. NA indicates no activity. ^∗∗∗∗∗^Greater than the lowest concentration of evaluated drugs. *R*^2^: coefficient of determination; values obtained from regression lines with 95% confidence level.

## Data Availability

The data used to support the findings of this study are included within the article or manuscript and the information mentioned is also included in the manuscript.
